# House and Home

**Published:** 1893-12

**Authors:** Lillian White


### HOUSE AND HOME.

CONDUCTED BY LILLIAN WHITE.

CHARITY BEGINS AT HOME.—We talk a lot about charity—let it begin in our own households. Respond to the missionary work that calls you right under your own roofs. Remember that you should do unto others as you would like to be done by, especially when the thermometer is up in the nineties, and if you wish to regard it as a business transaction you will find that you reap a greater reward in the end from this sort of dealing than by a system of suppression and repression that makes the relation of mistress and maid a most unpleasant one. Kindness to any fellow-being never comes amiss, and even though prompted by motives of selfish interest, the result proves quite as beneficial. Remember this, and either from innate goodness or the thought of better service in the future, show a genuine spirit of humanity to those who are in your employ.

FLOWERS IN THE HOUSE.—A tiny garden can be made by cutting a piece of sheet wadding to fit the top of a bowl or a wide-mouthed jar, which is filled with water just high enough for the bottom of the wadding to touch it. Two or three small bits of charcoal will keep the water pure, and, when all is arranged, the top of the wadding is sprinkled with seeds of mignonette, sweet pea or any other easily grown plant. The roots pierce down through the wadding and are nourished by the water, while leaves and blossoms, in a reasonable time, conceal the top. There is but one flower more beautiful than the morning-glory on the outside of the bay window, and that is a morning-glory trained up on the inside of the window, and in full bloom while the winter storms are raging and the thermometor is below zero. They can be grown with very little trouble.

DECORATED TRASH BASKETS.—Trash baskets have developed a decorative quality which makes them worthy of especial note. Two seen this past week are really striking handsome, and, as they are capacious as well, fill a definite want. One is tall and slender, but still firm upon its base, and is made entirely of heavy leather. The outside shows an embossed design that is in the best of taste and color. For an accompaniment to a library desk nothing better could be desired, and the many women who shortly will be seeking Christmas gifts for their husbands and friends will do well to bear it in mind. The other is more delicate, both in color and material, and is singularly appropriate for a lady's use. It is made of fine African grass and celluloid, and is dainty as can be, while at the same time it provides ample space.

### THE DRESS.

THE LATEST MODES.—Mink will be as popular as ever, both for garments and trimmings. Many fashionable capes and jackets will be made of Persian lamb or black marten.

Cuffs of lace or linen or muslin are again in vogue, the muslin ones being confined to woollen gowns.

Sleeves are enormous, but falling on the shoulders to the huge puff at the elbow. These are called the chatelaine sleeves.

Very pretty bonnets are close-fitting, with the coronet front closely flared back and cut in scallops or points, which are faced with velvet, embroidery, lace or spangles.

YELLOW IN THE DRESS.—A touch of yellow is given to many stylish wool dresses, as a full-pointed vest and piping of corn-yellow bengaline in a green cloth gown, or to brighten a black faille, cloth or satin dress. Chamois vests, collar and cuffs are added to day dresses of plaited or striped wool, with black serpentine braid as a finish. And French taste combines yellow and gray this season, the combination showing among new costumes made with a draped overskirt.

### THE KITCHEN.

DISHES FOR EVERYTHING.—There is a bewildering variety of dishes manufactured for special purposes; lovely salad bowls consist of geranium leaves in clouded browns and autumn leaf colors with sprays of pink or scarlet flowers. Charmingly cool looking and suggestive are the salad sets, the bowl formed of a hollowed head of cabbage; the plates are in the shape of a flat cabbage leaf. Elegant bowls are also of heavy faceted glass. Salad sets are ivory or boxwood forks and spoons, with handles of Dresden china or cut crystal.

SCALLOPED TURKEY.—Chop cold turkey, butter a dish, put a layer of bread crumbs in the bottom, then a layer of oysters; season with bits of butter, salt and pepper, then cover with a lawyer of turkey. Continue alternating in this way until the dish is full, having the last layer of crumbs. Pour over the whole a cream sauce made of one tablespoonful of butter, the same amount of flour and a cupful of cream or rich milk. Cover closely and bake in a moderate oven twenty minutes.

CRANBERRY SAUCE.—Wash and pick over one quart of selected cranberries, put them in a porcelain-lined stew-pan with half a pint of cold water and half a pound of granulated sugar. Boil for twenty minutes, stirring the berries as little as possible, in order that they may keep their shape. When cool the liquid will form a jelly about them.

CUSTARD PIE.—Beat two eggs without separating, add two teaspoonfuls of sugar, a pinch of salt and a little nutmeg. Scald two cups of creamy milk and pour on the eggs, stirring all the while. Strain into a deep pie-plate lined with paste. Bake slowly in a moderate oven, watching carefully. As soon as it puffs slightly on top and a knife blade comes out clean when run into the custard, it is done. The success of both custard and cream pies lies in the baking, as too long baking makes them watery.

LAMB À LA FRANÇAISE.—Put a leg of mutton in a kettle. Add, in a muslin bag, one small turnip, a few celery leaves, three sprays of marjoram and savory, four cloves and twelve allspice. Add two quarts of boiling water. Skim carefully. Simmer four hours. Add four large tablespoonfuls of flour smothered in water, salt, and a speck of cayenne. Take out the lamb and bag, skim and boil rapidly for ten minutes.

RYE BREAD.—Pour boiling water on rye flour or rye meal and mix into a stiff dough; make it into loaves about three inches in diameter, or cut into squares or rolls and bake in a hot oven.

ASBESTOS MATS.—One of the new kitchen improvements is a fire-proof mat to put on the table under a hot pot or kettle, a flat iron or an active gas-stove. These mats are about the size of a stove cover; they are made of asbestos, which is fire-proof; they are not much heavier than a dinner plate, and they sell at 7 and 10 cents each.

NEW ENGLAND PUDDING.—Heat three pints of milk to boiling and pour it over half a pint of yellow Indian meal salted with one tablespoonful of salt. Stir this very carefully, wetting it gradually so that there will be no lumps. Return it to the double boiler and cook slowly for forty minutes, with frequent stirrings. Stir in this butter the size of an egg, a cupful of molasses, one teaspoonful of ginger and one of mixed cinnamon and mace ; remove from the fire, heat hard and add slowly four well-whipped eggs and one cupful of seeded raisins. Butter a pudding dish, turn in the mixture and bake half an hour ; stir it from the bottom and finish the baking by browning nicely. Make a sauce with one cupful of powdered sugar, one tablespoonful of butter and one beaten egg; flavor with nutmeg.

